# Motion control and singular perturbation algorithms for lower limb rehabilitation robots

**DOI:** 10.3389/fnbot.2025.1562519

**Published:** 2025-05-09

**Authors:** Yanchun Xie, Anna Wang, Xue Zhao, Yang Jiang, Yao Wu, Hailong Yu

**Affiliations:** ^1^Department of Orthopaedics, General Hospital of Northern Theater Command, Shenyang, China; ^2^Department of Burns and Plastic Surgery, General Hospital of Northern Theater Command, Shenyang, China; ^3^Daniel L. Goodwin College of Business, Benedictine University, Chicago, IL, United States; ^4^Faculty of Robot Science and Engineering, Northeastern University, Shenyang, China

**Keywords:** rehabilitation robots, trajectory planning, singular perturbation, flexible control, controller design

## Abstract

To better assist patients with lower limb injuries in their rehabilitation training, this paper focuses on motion control and singular perturbation algorithms and their practical applications. First, the paper conducts an in-depth analysis of the mechanical structure of such robots and establishes detailed kinematics and dynamics models. An optimal S-type planning algorithm is proposed, transforming the S-type planning into an iterative solution problem for efficient and accelerated trajectory planning using dynamic equations. This algorithm comprehensively considers joint range of motion, speed constraints, and dynamic conditions, ensuring the smoothness and continuity of motion trajectories. Second, a zero-force control method is introduced, incorporating friction terms into the traditional dynamic equations and utilizing the LuGre friction model for friction analysis to achieve zero-force control. Furthermore, to address the multi-scale dynamic system characteristics present in rehabilitation training, a control method based on singular perturbation theory is proposed. This method enhances the system's robustness and adaptability by simplifying the system model and optimizing controller design, enabling it to better accommodate complex motion requirements during rehabilitation. Finally, experiments verify the correctness of the kinematics and optimal S-type trajectory planning. In lower limb rehabilitation robots, zero-force control can better assist patients in rehabilitation training for lower limb injuries, while the singular perturbation method improves the accuracy, response speed, and robustness of the control system, allowing it to adapt to individual rehabilitation needs and complex motion patterns. The novelty of this paper lies in the integration of the singular perturbation method with the LuGre friction model, significantly enhancing the precision of joint dynamic control, and improving controller design through the introduction of a torque deviation feedback mechanism, thereby increasing system stability and response speed. Experimental results demonstrate significant improvements in tracking error and system response compared to traditional methods, providing patients with a more comfortable and safer rehabilitation experience.

## 1 Introduction

With the global aging trend and evolving lifestyles, the demand for lower limb rehabilitation following injuries has surged, driving the widespread adoption of rehabilitation robots in medical applications. Notably, these robots exhibit significant potential in lower limb rehabilitation training (Qassim and Wan Hasan, 2020). Traditional rehabilitation relies heavily on specialized therapists to manually guide patients through passive exercises, a process that struggles to ensure consistency and continuity due to the limited availability of qualified therapists and variations in their skill levels stemming from individual differences. These factors represent key bottlenecks in current rehabilitation technology. Flexible lower limb rehabilitation robots address these challenges by simulating natural human movement patterns, offering personalized training programs, and facilitating the gradual restoration of motor functions, thereby alleviating the shortage of skilled trainers. However, technical challenges persist, particularly in achieving real-time responsiveness and precise trajectory planning in practical applications. The system models of lower limb rehabilitation robots typically exhibit complex characteristics, such as high order, non-linearity, and strong coupling. Additionally, the accuracy of these models is often challenging to ensure ultimately, which complicates the control structure. However, the singular perturbation method can simplify system models (Kevorkian and Cole, 2012) by partitioning the system into fast and slow subsystems based on time scales (Yu and Chen, 2015). Subsequently, controller design is conducted separately for these two subsystems. This approach reduces the order of the robot model and significantly decreases the computational burden. Li et al. (2021) developed an image-driven control strategy for the slow dynamic part involving rigid body motion to reduce visual errors, while compensating for errors in the approximated Jacobian matrix. For the fast dynamic part related to elastic vibrations, they designed an observer to predict the fast dynamic state to avoid relying on direct vibration state measurements. Based on these predicted states, a control feedback mechanism was developed to mitigate vibrations in the flexible robot. However, this system requires extra visual sensors, which introduces additional failure points. These visual sensors may experience malfunctions or degraded performance due to lighting conditions and dust.

The trajectory planning methods for rehabilitation robots have evolved from traditional robotic arms. Additionally, literature 0 introduced a smooth algorithm for trajectory planning of spray guns to address trajectory connection issues (From et al., 2010). Literature 0 explored the trajectory planning for the robot end effector (Dhanaraj et al., 2022). While literature 0 investigated the robot kinematic model using neural network approaches (Gao, 2020). However, due to the model complexity and the lengthy computation time, its engineering applications are limited. Motion control and singular perturbation control are critical technologies in rehabilitation robot control systems, directly impacting rehabilitation training effectiveness and safety. Further optimization of these control strategies significantly enhances the performance and clinical rehabilitation robot value.

This paper explores the development of kinematic and dynamic models for lower limb rehabilitation robots, providing a theoretical basis for trajectory planning. By applying maximum acceleration constraints to S-type trajectory planning, the study generates optimal acceleration trajectories that consider joint motion ranges, speed limits, and dynamic conditions, ensuring smooth and efficient robot operations. The integration of the LuGre friction model into the dynamic equations enables zero-force control, enhancing patient comfort during rehabilitation. Additionally, the combination of singular perturbation control and motor current-based joint torque control improves system performance, offering precise regulation and rapid response to dynamic changes. These advancements enhance the robustness and stability of the control system under external disturbances, supporting personalized rehabilitation treatments and expanding the clinical applicability of lower limb rehabilitation robots.

## 2 Motion control of lower limb rehabilitation robot

**Figure 1:**
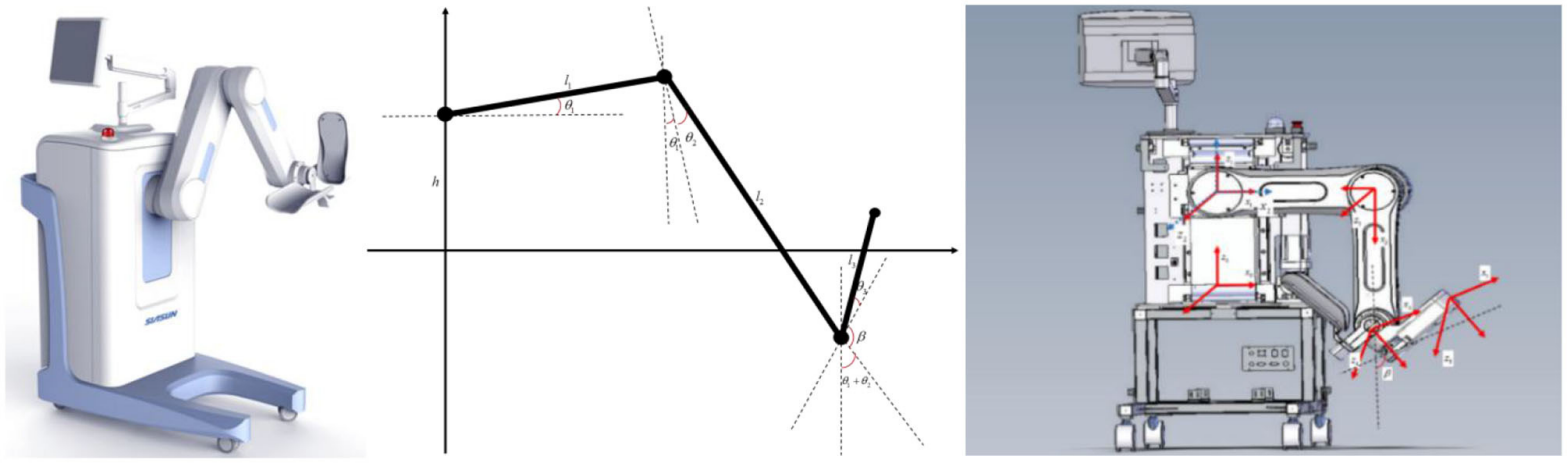
Coordinate system and connecting rod diagram of flexible lower limb rehabilitation robot.

As shown in Figure 1, this system employs a hierarchical open architecture design Based on the human lower limb structure and its regular movement mechanisms to enhance patients' efficiency and enjoyment during rehabilitation training while allowing for personalized adjustments based on individual differences. Regarding safety, the robot legs can be equipped with flexible tactile sensor modules, which enhance the robot's interactivity and the precision of rehabilitation training. The robot also incorporates multiple safety mechanisms, including force feedback, position limits, and emergency stop buttons, ensuring rapid response in abnormal situations to protect patient safety.

### 2.1 Kinematic model

In Figure 1, represent the lengths of the three links, while β denotes the angle between the extension line of link 3 and link 2. Let the height of the rise at joint 1 be *h*, and the rotational angles at the three joints be θ_1_, θ_2_, θ_3_. These three rotational angles collectively determine the spatial position and orientation. By adjusting the angles, the robot can navigate to different points in three-dimensional space, providing versatile functionality for patient interaction and treatment.

The DH parameters (as shown in Table 1) are used to describe the geometric relationship between adjacent joints in robotic manipulators, consisting of four key parameters: θ (joint angle), *d* (link offset), *a* (link length), and α (link twist angle). Specifically, θ represents the rotation angle about the *Z*-axis, describing the rotational displacement of the current joint and is typically a variable; *d* denotes the translation distance along the *Z*-axis, indicating the linear displacement of the current joint along the *Z*-axis and is usually a constant; *a* signifies the translation distance along the *X*-axis, representing the linear displacement of the current joint along the *X*-axis and is generally a constant; and α represents the rotation angle about the *X*-axis, describing the twist angle between adjacent joint axes and is typically a constant. Together, these parameters define the relative position and orientation between the joints of a robotic arm, providing a foundational framework for kinematic analysis.


(1) TH0= T10 · 21T · 32T · 43T· H4 T =[nxoxaxpxnyoyaypynzozazpz0001]

(2) $$\{\begin{array}{l} n_{x}=-C1S2C3\;+\;S1C2C\;3+\;C1C2S3\;+\;S1S2S3\\ n_{y}=0\\ n_{z}=S1S2C3\;-\;C1C2C3\;-\;S1C2S3\;-\;C1S2S3\\ o_{x}=C1S2S3\;-\;S1C2S3\;+\;C1C2C3\;+\;S1S2C3\\ o_{y}=0\\ o_{z}=0\\ a_{x}=0\\ a_{y}=-1\\ a_{z}=-S1S2S3\;+\;C1C2S3\;-\;S1C2C3\;-\;C1S2C3\\ p_{x}=l_{1}C1\;+\;l_{2}(-C1S2\;+\;S1C2)\;+\;l_{3}(-C1S2C3\;+\;S1C2C3\\ \;+\;C1C2S3\;+\;S1S2S3)\\ p_{y}=0\\ p_{z}=h-l_{1}S1\;+\;l_{2}(S1S2\;-\;CIC2)\;+\;l_{3}(S1S2C3-\\ C1C2C3\;-\;S1C2S3\;-\;C1S2S3) \end{array}$$

**Table 1:** Robot D-H parameters.

**rods**	**α**	**a**	**d**	**θ**
1	0	0	*h*	0
2	$\frac{\pi }{2}$	0	0	θ_1_
3	0	*l*_1_	0	$\theta_{2}+\frac{\pi }{2}$
4	0	*l*_2_	0	θ_3_+β
H	0	*l*_3_	0	0

The known variables are:


(3) $$\{\begin{array}{ll} \;C1=\cos\theta_{1} & \;\\ \;S1=\sin\theta_{1} & \;\\ \;C2=\cos\theta_{2} & \; \end{array}\{\begin{array}{ll} S2=\sin\theta_{2} & \;\\ C3=\cos\left(\theta_{3}+\beta \right) & \;\\ S3=\sin\left(\theta_{3}+\beta \right) & \; \end{array}$$

The pose transformation matrix (Equation 1) can be obtained according to DH table, and the forward kinematics equation can be derived. In this matrix, the first three columns correspond to the projections of the new coordinate system's *X*-axis, *Y*-axis, and *Z*-axis direction vectors within the original coordinate system, represented as $\text{n}={\left[n_{x},n_{y},n_{z}\right]}^{T}$ $\text{o}={\left[o_{x},o_{y},o_{z}\right]}^{T}$, respectively. These vectors collectively define the rotational (orientational) transformation of the new coordinate system relative to the original one. The fourth column $\text{p}={\left[p_{x},p_{y},p_{z}\right]}^{T}$ denotes the position vector of the new coordinate system's origin with respect to the original coordinate system, encapsulating the translational transformation. The final row [0, 0, 0, 1] serves as the normalization component of the homogeneous coordinates. Such transformation matrices are extensively utilized in robotic kinematics, where sequential multiplication facilitates the computation of the end-effector's pose relative to the base coordinate system.

### 2.2 Zero-force control

Set on the joint coordinates $q={\left[h\;q_{1}\;q_{2}\;q_{3}\right]}^{T}={\left[h\;\theta_{1}\;\theta_{1}\;+\;\theta_{2}\;\theta_{1}\;+\;\theta_{2}\;+\;\theta_{3}\right]}^{T}$. *m*_1_, *m*_2_ and *m*_3_ respectively, represent the mass of the three members, *l*_1_, *l*_2_ and *l*_3_ represent the length of the three members, *d*_1_, *d*_2_, *d*_3_ indicate the distance from the mass center to the axes of the three members, respectively.

(1) Rehabilitation of robot total potential energy

Potential energy *P*_1_ of rehabilitation robot rod 1


(4) $$\begin{array}{l} P_{1}=m_{1}g\left(h+d_{1}\cos q_{1}\right) \end{array}$$

The potential energy *P*_2_ of rehabilitation robot rod 2


(5) $$\begin{array}{l} P_{2}\;=\;m_{2}\;g\;\left(h\;+\;l_{1}\;\cos\;q_{1}\;-\;d_{2}\;\cos\;q_{2}\right) \end{array}$$

The potential energy *P*_3_ of rehabilitation robot rod 3


(6) $$\begin{array}{l} P=m_{3}g\left(h+l_{1}\sin q_{1}-l_{2}\sin q_{2}+d_{3}\cos\left(q_{3}+\beta \right)\right) \end{array}$$

The rehabilitation robot total potential energy as Equation 7.


(7) $$\begin{array}{l} P=P_{1}+P_{2}+P_{3} \end{array}$$

(2) The rehabilitation robot total kinetic energy of can be similarly obtained as follows


(8) $$\begin{array}{l} K=K_{1}+K_{2}+K_{3} \end{array}$$

The Lagrange function *L* is:


(9) $$\begin{array}{l} L=K-P=\left(K_{1}+K_{2}+K_{3}\right)-\left(P_{1}+P_{2}+P_{3}\right) \end{array}$$

The known variables are:


(10) $$\{\begin{array}{ll} \;C1=\cos q_{1} & \;\\ \;S1=\sin q_{1} & \;\\ \;C2=\cos q_{2} & \; \end{array}\{\begin{array}{ll} S2=\sin q_{2} & \;\\ C3=\cos\left(q_{3}+\beta \right) & \;\\ S3=\sin\left(q_{3}+\beta \right) & \; \end{array}$$

The driving torque for each joint of the rehabilitation robot is then given by:


(11) $$\begin{array}{l} \tau_{1}=\frac{d}{dt}\frac{\partial L}{\partial h}-\frac{\partial L}{\partial h}=\left(m_{1}+m_{2}+m_{3}\right)\ddot{h}+(m_{1}d_{1}+m_{2}l_{1}\\ +m_{3}l_{1})C1{\ddot{q}}_{1}+\\ (-m_{2}d_{2}+m_{3}l_{2})S2{\ddot{q}}_{2}-m_{3}d_{3}S3{\ddot{q}}_{3}-(m_{1}d_{1}+m_{2}l_{1}\\ +m_{3}l_{1})S1{\dot{q}}_{1}^{2}+\\ \left(-m_{2}d_{2}+m_{3}l_{2}\right)C2{\dot{q}}_{2}^{2}-m_{3}d_{3}C3{\dot{q}}_{3}^{2}-\left(m_{1}+m_{2}+m_{3}\right)g \end{array}$$

Similarly, we can get other joint driving torques. Take the friction term into the traditional dynamic equation and use the LuGre friction model for friction analysis and realize zero-force control. This study utilized a flexible lower-limb rehabilitation robotic experimental platform to approach enabled zero-force and high-precision control of the end effector force in the robot, achieving effective force feedback management.


(12) $$\begin{array}{l} M\left(q\right)\;\ddot{q}\;+\;C\left(q,\;\dot{q}\right)\;\dot{q}\;+\;G\left(q\right)\;+\;T_{f}\left(\dot{q}\right)\;=\;\tau \end{array}$$

*T*_*f*_is torque of LuGre friction, $\dot{q}$ is angular velocity vector, $\ddot{q}$ is rotational angular acceleration vector.

This paper uses the LuGre friction model to compensate for joint dynamics. LuGre friction method not only enhances the fit of friction characteristics simulation but also improves the overall accuracy of the collision detection model. The LuGre friction model is


(13) $$\begin{array}{l} T_{f}\left(\dot{q}\right)\;=\;\left\{\left[f_{C}\;+\;\left(f_{S}\;-\;f_{c}\right)\exp\left(-{\left(\frac{\overset{{}^{\circ}}{q}}{{\overset{{}^{\circ}}{q}}_{s}}\right)}^{2}\right)\right]\right\}\;sgn\left(\overset{{}^{\circ}}{q}\right)\;+\;\sigma_{2}\overset{{}^{\circ}}{q} \end{array}$$

In order to obtain the real friction parameters, experiments are carried out on the vacuum manipulator platform to obtain the following data: among others, *f*_*C*_ is Coulomb friction, *f*_*S*_ is static friction; $\dot{q}\left(t\right)$ provides real-time speed for the joints; ${\dot{q}}_{s}$ for Stribeck Speed δ is a parameter related to the shape of the contact surface; σ_2_ is the coefficient of viscous friction;τ(*t*) is the joint drive torque measured by a sensor. The sampling frequency is high enough to capture changes in friction over time.

In rehabilitation robotics, the LuGre friction model provides critical support for joint friction compensation. When a patient applies a minimal external force, the robot, based on real-time joint velocity measurements and identified friction parameters (such as static friction), generates an equivalent compensating torque through the controller to counteract the inherent friction resistance of the mechanical system. This process enables the end-effector to exhibit a “weightless” zero-force control effect for the patient, meaning that only a minimal force is required to drive the robot's motion. This significantly enhances the compliance of rehabilitation training and the safety of human-robot interaction. By experimentally calibrating parameters (e.g., low-speed sweep tests for static friction and high-speed constant velocity tests for viscous friction separation), the LuGre model further optimizes friction compensation accuracy, ensuring the robustness of zero-force control in complex motion scenarios.

### 2.3 Optimal S-type planning

Under optimal trajectory control, the lower limb rehabilitation robot significantly improves its trajectory planning ability and operational flexibility and maximizes the advantages of the lower limb rehabilitation robot. The trajectory interpolated core considers both kinematic constraints (such as joint angles and end-effector positions) and dynamic constraints (including forces, torques, and inertia). Optimal trajectory parameters that satisfy these constraints are computed using optimization algorithms.

A complete S-curve trajectory planning can be constructed based on Equations 14, 15.

*J*(*t*) is Jerk, *A*(*t*) is Acceleration and *V*(*t*) is Velocity:


(14) $$J(t)=\{\begin{array}{l} \;J0\leq t<t_{1}\\ 0\;t_{1}\;\leq \;t\;<\;t_{2}\\ -J\;t_{2}\;\leq \;t\;<\;t_{3}\\ 0\;t_{3}\;\leq \;t\;<\;t_{4}\\ -J\;t_{4}\;\leq \;t\;<\;t_{5}\\ 0\;t_{5}\;\leq \;t\;<\;t_{6}\\ J\;t_{6}\;\leq \;t\;<\;t_{7} \end{array}$$

$$A(t)=\left\{ \begin{array}{l} \;J\tau_{1}\;0\leq t<t_{1}\\ \;JT_{1}t_{1}\leq t<t_{2}\\ JT_{1}-J\tau_{3}t_{2}\leq t<t_{3}\\ \;0t_{3}\leq t<t_{4}\\ \;-J\tau_{5}t_{4}\leq t<t_{5}\\ \;-JT_{5}t_{5}\leq t<t_{6}\\ -JT_{5}+J\tau_{7}t_{6}\leq t<t_{7} \end{array}\right.$$

$$V(t)=\{\begin{array}{c} V_{s}+\frac{1}{2}J\tau_{1}^{2}0\leq t<t_{1}\\ V_{01}+JT_{1}\tau_{2}t_{1}\leq t<t_{2}\\ V_{02}+JT_{1}\tau_{3}-\frac{1}{2}J\tau_{3}^{2}t_{2}\leq t<t_{3}\\ V_{03}t_{3}\leq t<t_{4}\\ V_{04}-\frac{1}{2}J\tau_{5}^{2}t_{4}\leq t<t_{5}\\ V_{05}-JT_{5}\tau_{6}t_{5}\leq t<t_{6}\\ V_{06}-JT_{5}\tau_{7}-\frac{1}{2}J\tau_{7}^{2}t_{6}\leq t<t_{7} \end{array}$$

Position is *S*(*t*):


(15) $$S(t)=\{\begin{array}{l} V_{s}\tau_{1}+\frac{1}{6}J\tau_{1}^{3}\;0\leq t<t_{1}\;\\ S_{01}+V_{01}\tau_{2}+\frac{1}{2}JT_{1}\tau_{2}^{2}\;t_{1}\leq t<t_{2}\;\\ S_{02}+V_{02}\tau_{3}+\frac{1}{2}JT_{1}\tau_{3}^{2}-\frac{1}{6}J\tau_{3}^{2}\;t_{2}\leq t<t_{3}\;\\ S_{03}+V_{03}\tau_{4}\;t_{3}\leq t<t_{4}\;\\ S_{04}+V_{04}\tau_{5}-\frac{1}{6}J\tau_{5}^{2}\;t_{4}\leq t<t_{5}\;\\ S_{05}+V_{05}\tau_{6}-\frac{1}{2}JT_{5}\tau_{6}^{2}\;t_{5}\leq t<t_{6}\;\\ S_{06}+V_{06}\tau_{7}-\frac{1}{2}JT_{5}\tau_{7}^{2}+\frac{1}{6}J\tau_{7}^{3}\;t_{6}\leq t<t_{7}\; \end{array}$$

An example of a planning segment from time node *t*_0_to *t*_7_is considered, where *t*_0_ represents the initial moment and *t*_7_ indicates the final moment. At both the start and end points, the robot velocity and acceleration are set to zero to ensure a smooth initiation and cessation of motion. Furthermore, it is essential to account for the limits of robot speed, acceleration, and jerk (the accelerated change rate) during the movement process. These include the max velocity *v*_*m*_, the max acceleration *a*_*mu*_, the max deceleration *a*_*md*_, as well as the max jerk during the acceleration and deceleration phases, denoted as *j*_*mu*_, *j*_*md*_. These parameters are directly related to robot safety and efficiency.

The states of trajectory planning at each discrete point $\left(q_{i},{\dot{q}}_{i},{\ddot{q}}_{i}\right)$ are obtained using the robot S-type programming model. Based on the established dynamic model (Equation 16), the inverse dynamic IDy is used to solve the theoretical torque τ_*i, cal*_ of each joint demand.


(16) $$\begin{array}{l} \tau_{i,cal}=IDy\left(q_{i},{\dot{q}}_{i},{\ddot{q}}_{i}\right) \end{array}$$

The calculated moment value is compared with each manipulator joint's maximum output moment τ_*i*, max_. If the calculated moment value exceeds the theoretical moment value, it indicates that the joint cannot provide the moment value and must be corrected to make the planning result conform to the moment limit. The corrected joint calculated moment is as follows Equation 17.


(17) $$\begin{array}{l} \tau_{i,\text{calmax}}=\mathrm{sign}\left(\tau_{i,cal}\right)\cdot \min\left(\tau_{i,\max},|\tau_{i,\text{cal}}|\right) \end{array}$$

The optimal trajectory control can be achieved only when the robot dynamics constraint is applied to the trajectory planning of the manipulator's end. According to the dynamic equation of the manipulator, the corresponding relationship between the joint torque and acceleration ${\ddot{q}}_{\text{newmax}}$ is obtained as follows Equation 18.


(18) $$\begin{array}{l} {\ddot{q}}_{\text{newmax}}=M\left(q\right)\left(\tau_{i,\text{calmax}}-{\dot{q}}^{T}C\left(q\right)\dot{q}-g\left(q\right)\right) \end{array}$$

On this basis, the torque constraint is mapped to the acceleration constraint, the acceleration subject to the torque constraint is obtained, and the final acceleration constraint is obtained by comparing it with the joint acceleration in the initial state. The process is as follows Equation 19. ${\ddot{q}}_{i,\max}$ is the maximum acceleration constraint in the initial state, ${\ddot{q}}_{i,\text{modified}}$ is the maximum acceleration of the *i* joint calculated by torque constraint.


(19) $$\begin{array}{l} {\ddot{q}}_{i,\text{newmax}}=\min\left({\ddot{q}}_{i,\max}|{\ddot{q}}_{i,\text{modified}}|\right) \end{array}$$

${\ddot{q}}_{i,newmax}\;$is the maximum acceleration constraint adjusted by the dynamic constraint, and $\ddot{q}$ is the vector formed by the maximum joint acceleration calculated by the torque constraint. If the new acceleration constraint is substituted into the dynamic trajectory planning, the complete trajectory planning satisfies the dynamic constraint.

## 3 Singular perturbation method of lower limb rehabilitation robot

In the widespread robot application, a particular type of system is often encountered, characterized by significant differences in the rates of change between various states, exhibiting unique singularities and separated phenomena in motion (Weingartshofer et al., 2023; Amersdorfer and Meurer, 2022; Jiang et al., 2023; Wang et al., 2021; Pana et al., 2023; Cao et al., 2023; Zhang et al., 2024). Such systems, including electric motors, generators, precision robotic structures, and complex biological systems, are collectively called singularly perturbed systems or systems with dual time scales. For these dual time-scale dynamic systems, also known as singular perturbation systems, the design of control strategies must consider their multi-time-scale characteristics. Traditional control methods based on a single time scale are often inadequate, as the dynamic behaviors of such systems differ significantly across various time scales. To achieve precise control of these systems, it is common to utilize singular perturbation theory to decompose the original system into multiple subsystems that are temporally separated yet mutually coupled, with each subsystem corresponding to a specific time scale.

### 3.1 Singular perturbation model of robotics

This chapter focuses on transforming dynamic models into singular perturbation forms. Based on the inherent dynamic characteristics, mathematical transformation methods are applied to derive robot system models that comply with the requirements of singular perturbation theory analysis. During this derivation process, friction factors are temporarily excluded from consideration to simplify the analysis and highlight the core dynamic characteristics. The aim is to capture the essential dynamic behavior, it provides a theoretical framework for the design of subsequent controllers based on singular perturbation theory. The singular perturbation model is categorized into separate fast and slow subsystems. Typically, the fast subsystem reflects the high-frequency dynamic characteristics, while the slow subsystem characterizes the long-term behavior and primary motion trends.

By temporarily neglecting the influence of friction, a simplified dynamic model of the robotic joint torque system can be expressed as follows Equation 20, *N* is the accelerator transmission ratio, *J*_*em*_ is motor rotor inertia, is the electromotor torque formula, which from the AC permanent magnet synchronous motor torque model. τ is joint torque, τ_*ext*_ is the external reaction torque.


(20) $$\{\begin{array}{l} M(q)\ddot{q}+C(\dot{q},\dot{q})\dot{q}+G(q)=\tau +\tau_{ext}\\ NJ_{em}\ddot{q}+\tau =N\tau_{em}\\ \tau_{em}=\frac{3}{2}n_{p}y_{f}i_{sq} \end{array}$$

It can be observed as follows Equation 21.


(21) $$\begin{array}{l} \tau =\frac{3}{2}Nn_{p}\psi_{f}i_{sq}-NJ_{em}\frac{d\omega }{dt} \end{array}$$

In the analysis of robotic dynamics, the electromotor torque τ_*em*_ serves as the driving source, and its output is transmitted to the robot joints through the transmission system, resulting in the joint torque τ. When the robot design includes joint torque sensors, this critical parameter can be directly measured. The expression for joint torque is given by Equation 22, where *K* represents the inherent stiffness of the joint, *N*^−1^θ is the equivalent joint angular displacement obtained by inversely transforming the motor angle θ through the gear ratio *N*, and *q* is the actual joint angle. This Equation illustrates the dynamic relationship between joint torque, motor angle, and joint angle, as well as the impact of stiffness *K* on system response. The motor angular acceleration is obtained from Equation 23.


(22) $$\begin{array}{l} \tau =K\left(N^{-1}\theta -q\right) \end{array}$$

(23) $$\begin{array}{l} \ddot{\theta }=NK^{-1}\ddot{\tau }+N\ddot{q} \end{array}$$

Substituting Equations 22, 23 into the second equation in Equation 20, we get Equation 24.


(24) $$\begin{array}{l} N^{2}J_{m}K^{-1}\ddot{\tau }+\tau =N\tau_{m}-N^{2}J_{m}\ddot{q} \end{array}$$

Since the inertia matrix is a symmetric positive definite matrix, the following equation can be obtained.


(25) $$\begin{array}{l} \ddot{q}=M{\left(q\right)}^{-1}\left(\tau +\tau_{\text{ext}}-C\left(q,\dot{q}\right)\dot{q}-G\left(q\right)\right) \end{array}$$

Substituting Equation 25 into Equation 24 gives Equation 26.


(26) $$\begin{array}{l} K^{-1}\ddot{\tau }+\left(J_{m}^{-1}N^{-2}+M{\left(q\right)}^{-1}\right)\tau =J_{m}^{-1}N^{-1}\tau_{m}+M{\left(q\right)}^{-1} \end{array}$$

$$\begin{array}{l} \;\left(C\left(q,\dot{q}\right)\dot{q}+G\left(q\right)-\tau_{ext}\right) \end{array}$$

By introducing a small parameter ε, the system equations can be reformulated as a standard singular perturbation model. The joint stiffness *K* is often very high, so $\frac{K_{\varepsilon }}{\varepsilon^{2}}$ can be used instead of *K*. *K*_ε_ is positive definite diagonal matrix. Substituting the expression of *K* and sorting it out, we can get the singular perturbation model of the system:


(27) $$\{\begin{array}{l} M(q)\ddot{q}+C(\dot{q},\dot{q})\dot{q}+G(q)=\tau +\tau_{\text{ext }}\\ \varepsilon^{2}\ddot{\tau }+K_{\varepsilon }\left(J_{em}^{-1}N^{-2}+M{\left(q\right)}^{-1}\right)\tau =K_{\varepsilon }J_{em}^{-1}N^{-1}\tau_{em}\\ +K_{\varepsilon }M{\left(q\right)}^{-1}\left(C(q,\dot{q})\dot{q}+G(q)-\tau_{\text{ext }}\right) \end{array}$$

The system boundary layer model is derived by introducing a new coordinate variable as follows Equation 28.


(28) $$\{\begin{array}{l} h(q,\dot{q},t)={\left(J_{em}^{-1}N^{-2}+M^{-1}\right)}^{-1}(J_{em}^{-1}N^{-1}{\bar{\tau }}_{em}\\ +M^{-1}(C\dot{\bar{q}}+G-{\bar{\tau }}_{\text{ext }}))\\ \gamma =\tau -h(q,\dot{q},t),y\in {\mathbb{R}}^{n} \end{array}$$

Introduce a new time scale as follows Equation 29.


(29) $$\{\begin{array}{l} v=(t-t_{0})/\varepsilon \\ \text{if }\varepsilon =0 \end{array}$$

Equation 30 is derived from Equations 28, 29.


(30) $$\begin{array}{l} \;\frac{d^{2}\gamma }{dt^{2}}+K_{s}\left(J_{em}^{-1}N^{-2}+M^{-1}\right)\left(\gamma +h\right) \end{array}$$

$$\begin{array}{l} =K_{s}{J_{em}}^{-1}N^{-1}\tau_{em}+K_{s}M^{-1}\left(C\dot{q}+G-\tau_{ext}\right) \end{array}$$

At the time scale *v*, the state variables and the external inputs τ_*ext*_ are regarded as static values. Equation 31 can be derived.


(31) $$\begin{array}{l} \left({J_{em}}^{-1}N^{-2}+M^{-1}\right)h={J_{em}}^{-1}N^{-1}{\bar{\tau }}_{em} \end{array}$$

$$\begin{array}{l} \;+M^{-1}\left(C\dot{q}+G-{\bar{\tau }}_{\text{ext}}\right) \end{array}$$

By substituting it into Equation 30, the final system boundary layer model is as follows Equation 32.


(32) $$\begin{array}{l} \frac{d^{2}y}{dv^{2}}+K_{s}\left(J_{em}^{-1}N^{-2}+M^{-1}\right)\gamma =K_{s}J_{em}^{-1}N^{-1}\left(\tau_{em}-{\bar{\tau }}_{em}\right) \end{array}$$

### 3.2 Controller design based on singular perturbation

**Figure 2:**
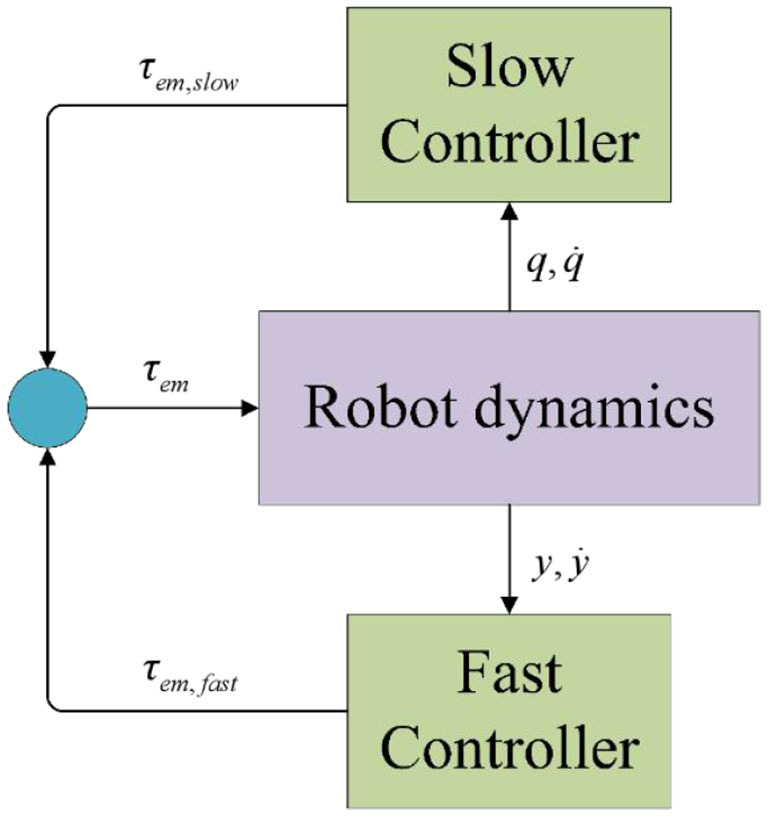
Singular control structure diagram.

In robotic controller design, singular perturbation methods are often integrated into composite feedback strategies to optimize system performance, as shown in Figure 2. This decomposition strategy independently addresses the different time scale issues in the system dynamics, thereby enhancing system stability, improving response speed, and simplifying the controller complexity (Bhardwaj et al., 2021; Khan et al., 2022; Zheng et al., 2022; Iskandar et al., 2022).

This design decomposes the control input into slow and fast system components, as shown in Equation 33.


(33) $$\begin{array}{l} \tau_{em}=\tau_{em,slow}+\tau_{em,fast} \end{array}$$

The slow subsystem represents the long-term behavior and primary motion trends of the robot. It is characterized by slower dynamics, such as the robot's overall trajectory and position control. The slow subsystem employs classical control strategies, such as computed torque control and torque feedforward control, which are commonly used in rigid robot models. These strategies ensure that the robot's motion is smooth and accurate over time.

For the selection of the control input τ_*em, slow*_ for the slow subsystem, it must be based on the assumption that the system has a standard form, meaning that the average time scale $\bar{\tau }$ in the quasi-steady-state model exists and has a unique solution. The key point is that the determination of τ_*em, slow*_ should depend solely on the state variables of the quasi-steady-state model $\left(q,\dot{q}\right)$, avoiding the direct introduction of instantaneous feedback from the joint torque τ, to ensure the independence and stability of the control input.

The fast subsystem captures high-frequency dynamics, such as vibrations and rapid adjustments in the robot's joints. These dynamics are critical for ensuring stability during rapid movements or when external disturbances are present.

In designing the control input τ_*em, fast*_ for the fast subsystem, it is essential to strictly adhere to the principle that it has no impact on the quasi-steady-state model when boundary layer effects are significant (ε = 0). This requires that the norm tends to zero under the limit condition (τ_*em, fast*_|_ε*to*0_ = 0). This design criterion aims to isolate potential disturbances from the fast subsystem on the dynamic behavior of the slow subsystem, ensuring the overall stability and accuracy of the system during rapid dynamic adjustments while optimizing performance in the boundary layer region. And we get the Equation 34.


(34) {τem,slow=τem|calE→0=τ¯τem,fast=τem−τ−=−εDτγ˙

It becomes clear that this structure aligns with that of a rigid robot model. This alignment allows for the direct application of classical control strategies used for rigid manipulators, such as the computed torque method and torque feedforward control, for design slow subsystem.

The damping matrix *D*_τ_ is designed as a positive definite matrix to ensure the stability and convergence of the system. A positive definite damping matrix introduces an energy dissipation mechanism into the system, which helps to suppress oscillations and stabilize the system. This is particularly crucial for rehabilitation robots, as smooth and stable motion is vital for patient safety and comfort.

For the slow subsystem, the controller design follows the fundamental principles (Equation 35) of the rigid manipulator model. The variables $q,\dot{q}$ represent the joint position and velocity. Considering the specificity of the boundary layer system, where time *t* and state variables can be treated as constants within a specific analytical framework, the original term $-\varepsilon D_{\tau }\dot{\gamma }$ can be reasonably transformed into $-\varepsilon D_{\tau }\dot{\tau }$. This transformation deepens the understanding of the system dynamic behavior under boundary conditions.


(35) $$\begin{array}{l} \tau_{em,slow}=\tau_{rigid}\left(q,\dot{q}\right) \end{array}$$

The design of the overall composite feedback controller for the robotic system integrates the control strategies of the aforementioned fast and slow subsystems. By adjusting the positive definite damping matrix, the control laws of the rigid manipulator model, and the dynamic adjustments under boundary layer effects, a robust and efficient control system framework is ultimately formed. This framework is capable of addressing complex and variable operational environments and task requirements.


(36) $$\begin{array}{l} \tau_{em}=\tau_{\text{rigid}}\left(q,\dot{q}\right)-\varepsilon D_{\tau }\dot{\tau } \end{array}$$

It utilizes an increased damping term to act on the system. However, it does not address or optimize the dynamic characteristics of the boundary layer, which limits the system's overall performance improvement. To overcome this shortcoming, we introduce a torque deviation feedback mechanism. By monitoring and feeding back torque deviations in real-time, we can dynamically adjust the control strategy, enhancing system stability and significantly improving the boundary layer's dynamic response and adjustment capability, leading to more efficient system control.

The torque deviation feedback is Equation 37.


(37) $$\begin{array}{l} K_{\tau }\gamma =K_{\tau }\left(\tau -h\left(q,q,t\right)\right) \end{array}$$

*K*_τ_is gain matrix, which used to improve the boundary layer dynamic performance. Therefore, the entire controller can be expressed as follows:


(38) $$\begin{array}{l} \tau_{em}=\tau_{\text{rigid}}\left(q,\dot{q}\right)-K_{\tau }\gamma -\varepsilon D_{\tau }\dot{\tau }=\tau_{\text{rigid}}\left(q,\dot{q}\right) \end{array}$$

$$\begin{array}{l} \;-K_{\tau }\left(\tau -h\left(q,\dot{q},t\right)\right)-\varepsilon D_{\tau }\dot{\tau } \end{array}$$

### 3.3 Improved controller design

To implement an effective feedback mechanism, it is often necessary to accurately solve the steady-state function $h\left(q,\dot{q},t\right)$. However, this can be quite challenging in practical applications, such as the difficulties in accurately measuring external torque τ_ext_ and the complex computation of the inverse inertia matrix *M*(*q*). The Tychonov theorem states that for trajectory tracking errors, it ensures that the deviation from the quasi-steady state is bounded by a high-order infinitesimal quantity ε. This characteristic becomes significant only as ε approaches zero, highlighting the remarkable advantage of the boundary layer model over the quasi-steady state model in terms of dynamic response speed. By finely tuning the parameter ε, rapid convergence of the error to a range close to the quasi-steady state can be achieved. Its introduction enables us to decompose complex multi-timescale systems into two independent subsystems—the fast subsystem and the slow subsystem—thereby simplifying controller design. The fast subsystem is typically employed to address high-frequency dynamics (e.g., vibrations, rapid adjustments), while the slow subsystem is utilized to manage low-frequency dynamics (e.g., overall trajectory control, position control).

To lessen the complexity of the state function, the controller is broken down into the following structure.


(39) $$\begin{array}{l} \tau_{em}=\tau_{ss}\left(q,\dot{q}\right)-K_{\tau }\tau -\varepsilon D_{\tau }\dot{\tau } \end{array}$$

The positive definite control gain matrix *K*_τ_, *D*_τ_ are crucial, as they ensure the system stability and responsiveness. The real-time control input $\tau_{ss}\left(q,\dot{q}\right)$ in the quasi-steady state model is dynamically adjusted based on the system state to achieve precise control. By replacing *K*_τ_τ in the original Equation 39 with *K*_τ_*y*, it directly affects the dual time scale control strategy. On one hand, it directly acts on the fast system component, optimizing the computation of τ_*m, fast*_ to enhance the system rapid response capability and dynamic performance. On the other hand, this strategy also indirectly affects the control input of the slow system component τ_*m, slow*_ by optimizing the long-term control strategy, thereby improving the system robustness and steady-state accuracy. During the design process, careful adjustments to *K*_τ_, *D*_τ_ are necessary, taking into account the interactions between the fast and slow systems to ensure that the overall control system can respond quickly to external changes while maintaining long-term stable operation.


(40) $$\begin{array}{l} \tau_{ss}\left(\bar{q},\bar{q}\right)=N^{-1}\left(I+K_{\tau }\right)\tau_{d},\tau_{d} \end{array}$$

(41) $$\begin{array}{l} \left(M\left(\bar{q}\right)+{\left(I+K_{\tau }\right)}^{-1}N^{2}J_{em}\right)\bar{\bar{q}}+C\left(\bar{q},\bar{q}\right)\bar{q}+G\left(\bar{q}\right) \end{array}$$

$$\begin{array}{l} \;={\left(I+K_{\tau }\right)}^{-1}N\tau_{ss}\left(\bar{q},\bar{q}\right) \end{array}$$

τ_*d*_as a new control input, it can be designed based on rigid robots to derive the steady-state system as follows Equation 41. Finally, we get the Equation 42 and Closed-loop control system Equation 43.


(42) $$\begin{array}{l} \tau_{em}=\tau_{d}-K_{\tau }\left(\tau -\tau_{d}\right)-\varepsilon D_{\tau }\dot{\tau } \end{array}$$

(43) $$\begin{array}{l} \;\frac{d^{2}\gamma }{dv^{2}}+K_{\epsilon }J_{em}^{-1}N_{-1}D_{\tau }\frac{d\gamma }{dv}+k_{\epsilon }J_{em}^{-1} \end{array}$$

$$\begin{array}{l} \left(N^{-2}+J_{em}M{\left(q\right)}^{-1}+N^{-1}K_{\tau }\right)\gamma =0 \end{array}$$

For the newly constructed quasi-steady state system, the design of the inertia matrix *K*_τ_ is closely related to the control gain matrix. We can dynamically configure the inertia distribution coefficient of the motor rotor by adjusting this parameter. This not only enhances the system resistance to external disturbances but also optimizes the response speed and stability of the control loop.

For the modified boundary layer system, the control input for the fast subsystem part is in the form of Equation 44.


(44) $$\begin{array}{l} \tau_{em,\text{fast}}=-K_{\tau }\left(\bar{\tau }-\bar{\tau }\right)-\varepsilon D_{\tau }\dot{\tau }=-K_{\tau }\gamma -\varepsilon D_{\tau }\dot{\tau } \end{array}$$

(45) $$\begin{array}{l} \frac{d^{2}\gamma }{dv^{2}}+K_{\varepsilon }J_{em}^{-1}N^{-1}D_{\tau }\frac{d\gamma }{dv}+K_{\varepsilon }J_{em}^{-1} \end{array}$$

$$\begin{array}{l} \left(N^{-2}+J_{em}M{\left(q\right)}^{-1}+N^{-1}K_{\tau }\right)\gamma =0 \end{array}$$

## 4 Experiment

### 4.1 Kinematics and trajectory planning

The kinematic experiment uses cylindrical helices and conical helices to verify the kinematic trajectories. The parametric equation for a cylindrical helix can be expressed as Equation 46.


(46) $$\{\begin{array}{l} x(t)=R\;\cdot \;\cos(t)\\ y(t)=R\;\cdot \;\sin(t)\\ z(t)=c\;\cdot \;t \end{array}$$

Here, *t* is the parameter, *R* is the radius of the helix, and *c* is the pitch constant, which represents the distance the helix moves in the *z* direction for each complete turn.

The parametric equation for a conical helix can be expressed as Equation 47.


(47) $$\{\begin{array}{l} x(t)=\left(R_{0}+kt\right)\;\cdot \;\cos(t)\\ y(t)=\left(R_{0}+kt\right)\;\cdot \;\sin(t)\\ z(t)=c\;\cdot \;t \end{array}$$

Here, *R*_0_ is the initial radius of the helix, *k* is the pheasant rate of change and *c* is the pitch constant.

**Figure 3:**
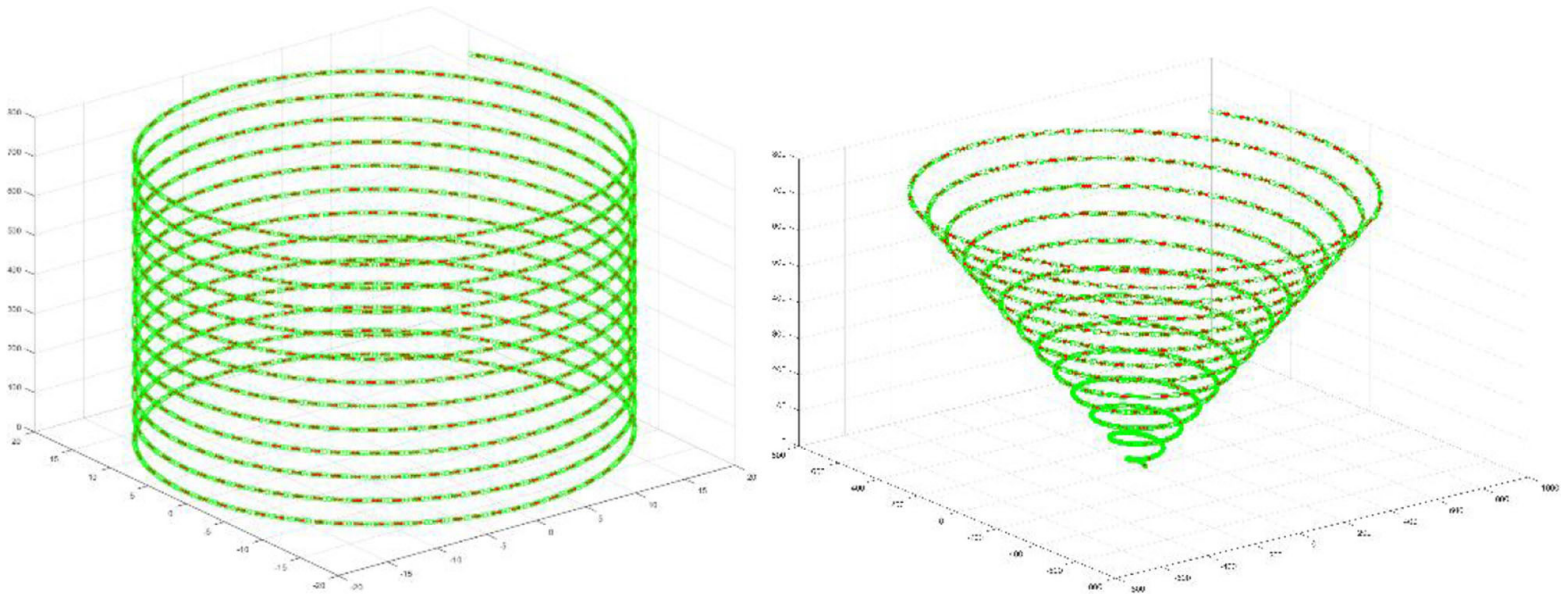
Cylindrical helix and conical helix.

As shown in Figure 3, by comparing the original end effector trajectory with the trajectory obtained from the forward kinematics calculations, the results indicate a high degree of consistency between the two. This not only verifies the correctness of the inverse and forward solution algorithms but also further demonstrates the accuracy and reliability of the robot kinematic model in design and practical applications.

**Figure 4:**
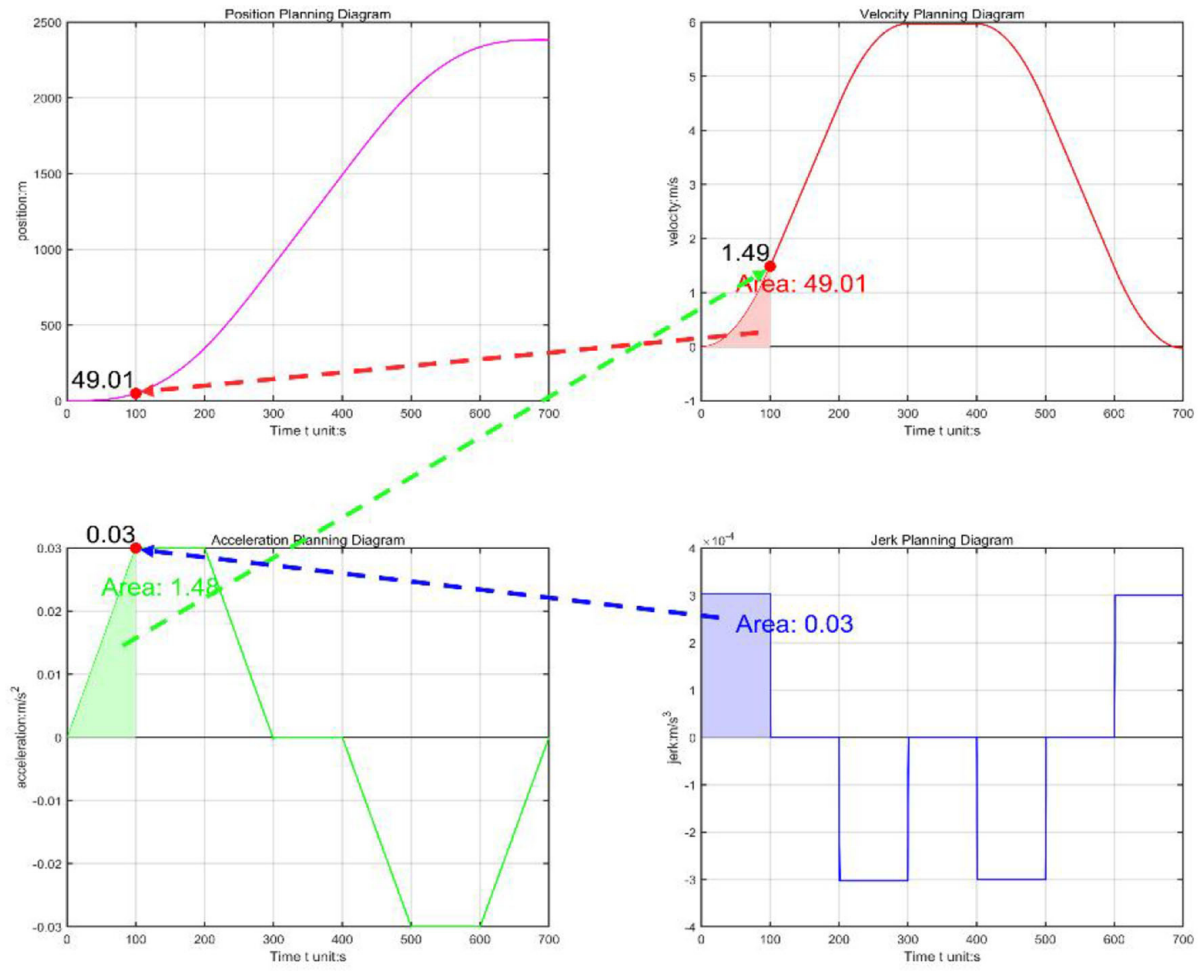
S-type trajectory planning.

Figure 4 clearly illustrates the trajectory constraints of S-curve trajectory planning within a specified time frame and how to accurately generate trajectories that comply with preset acceleration limits, thereby validating the correctness of this planning method. The main advantage of trajectory planning in joint space is the direct control of the robotic joint movements. Compared to trajectory planning in operational space, this approach significantly enhances operational flexibility and accuracy. Furthermore, this method effectively avoids potential issues arising from motion singularities and operational complexities, thereby enhancing the robustness of the system.

**Figure 5:**
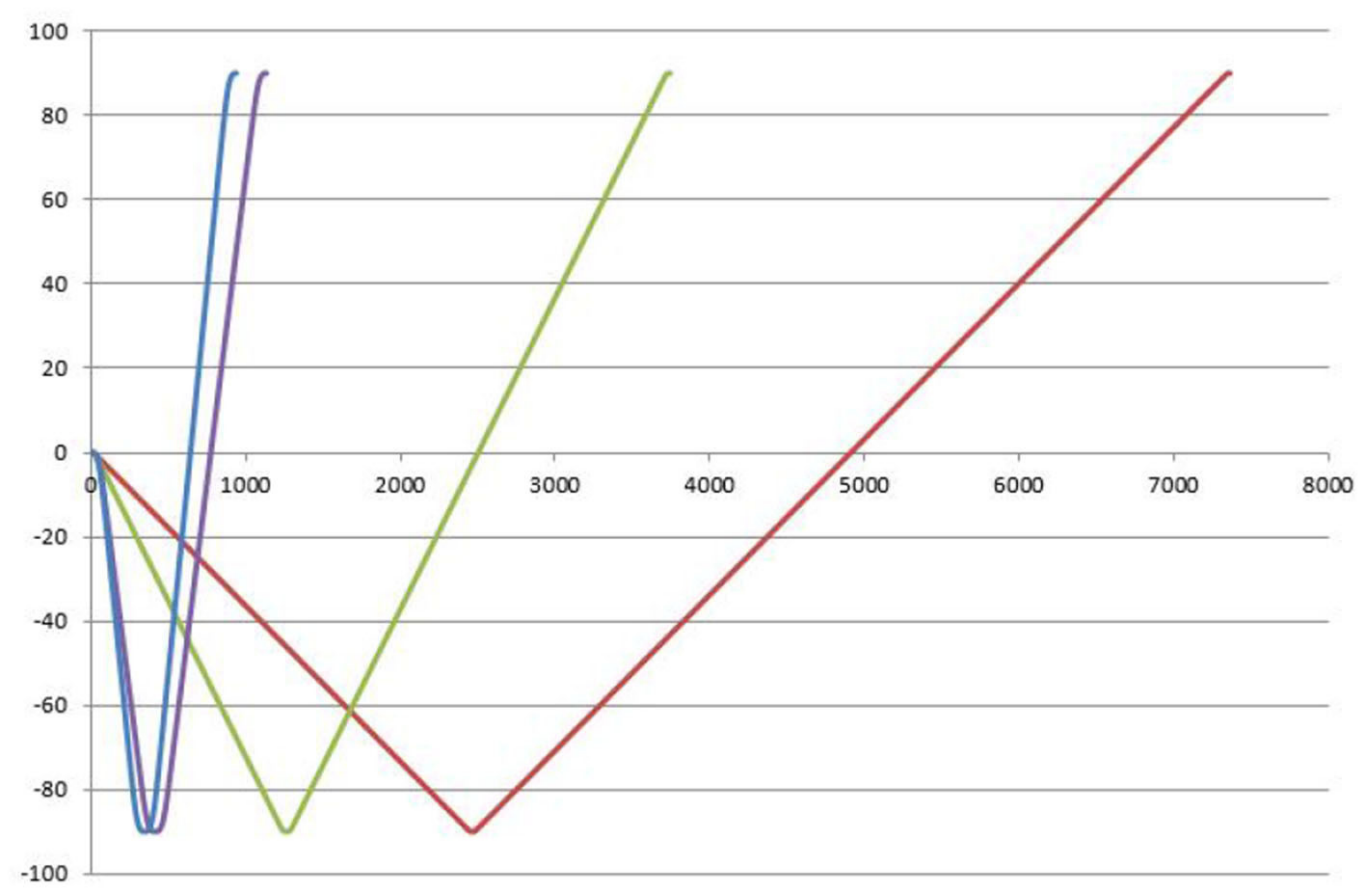
Optimal trajectory control end position time.

In this paper, the torque value obtained by the complete dynamic model is converted into the acceleration value, and the acceleration value is further applied as a limiting factor in trajectory planning to obtain the optimal trajectory control of the lower limb rehabilitation robot. As shown in Figure 5, the horizontal coordinate is the time, and the vertical coordinate is the TCP end position of the vacuum manipulator. Red is the general planning curve, and blue is the optimal trajectory curve. When the end position is from 0 to −80 and then to 90, the robot's time is obtained from the acceleration calculated by the dynamics, and the planning time becomes significantly faster.

The acceleration calculated by the dynamics is applied to the S-type trajectory planning, and the optimal trajectory control of the acceleration of the S-type trajectory is performed indirectly according to the acceleration solved by the dynamics to achieve the corresponding optimal. Comparing the running time with the ordinary planning time, optimal S-type planning performs quickly. Optimal S-type planning typically requires that servo drives use position control, which enhances the speed and current response of the motor, thereby improving the effectiveness of position control.

### 4.2 Zero-force control based on friction model

**Figure 6:**
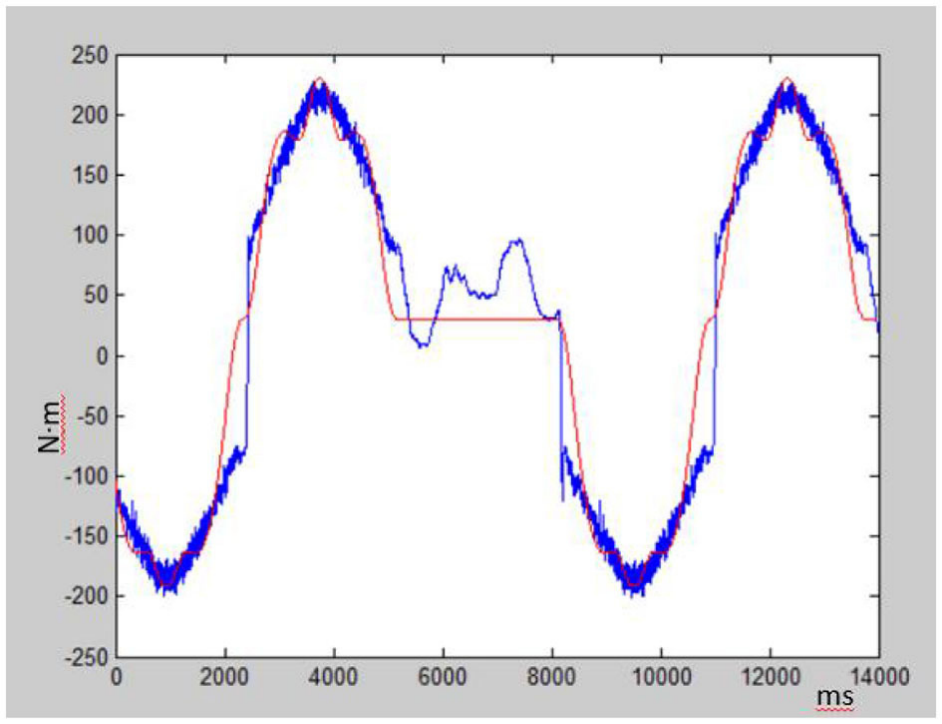
Comparison of actual torque and theoretical torque on zero-load control.

Zero force control is a compliant control strategy that aims to ensure that the force between the system and the environment remains at zero level. The LuGre friction parameters are identified and optimized, and the theoretical driving torque is solved against the collected actual torque, as shown in Figure 6. The blue curve represents the real friction moment curve, while the red curve is the moment curve of the LuGre friction model proposed in this paper. The deviation of actual friction and LuGre friction is calculated to be about 5 Nm. The results verify that the whole dynamic algorithm with The LuGre friction can effectively solve zero-force control.

**Figure 7:**
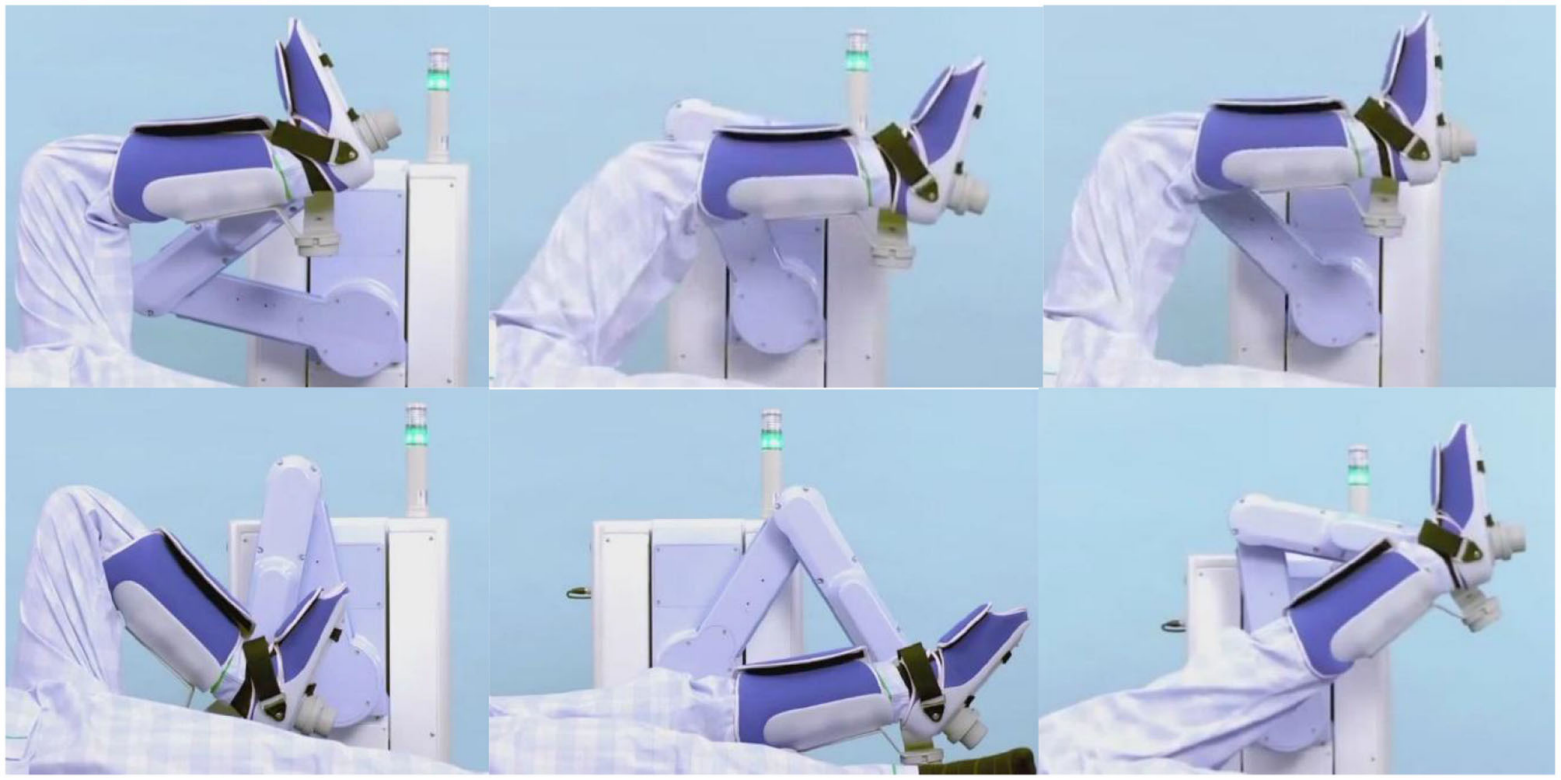
Zero-load control of flexible lower limb rehabilitation robot.

To intuitively and scientifically validate the effectiveness of the dynamic algorithm, this study utilized a flexible lower-limb rehabilitation robotic experimental platform to demonstrate a zero-load control algorithm. As shown in Figure 7, this approach enabled high-precision control of the end effector force in the robot, achieving effective force feedback management.

Implementing the zero-load control algorithm on the experimental platform signifies that the robot can more accurately simulate the natural movement patterns of the human body while performing rehabilitation training tasks. This reduces unnecessary mechanical resistance and discomfort, enhancing patient rehabilitation experience and therapeutic outcomes. Such advancements have profound implications for improving rehabilitation efficiency, shortening recovery periods, and reducing overall rehabilitation costs.

### 4.3 Singular perturbation

**Figure 8:**
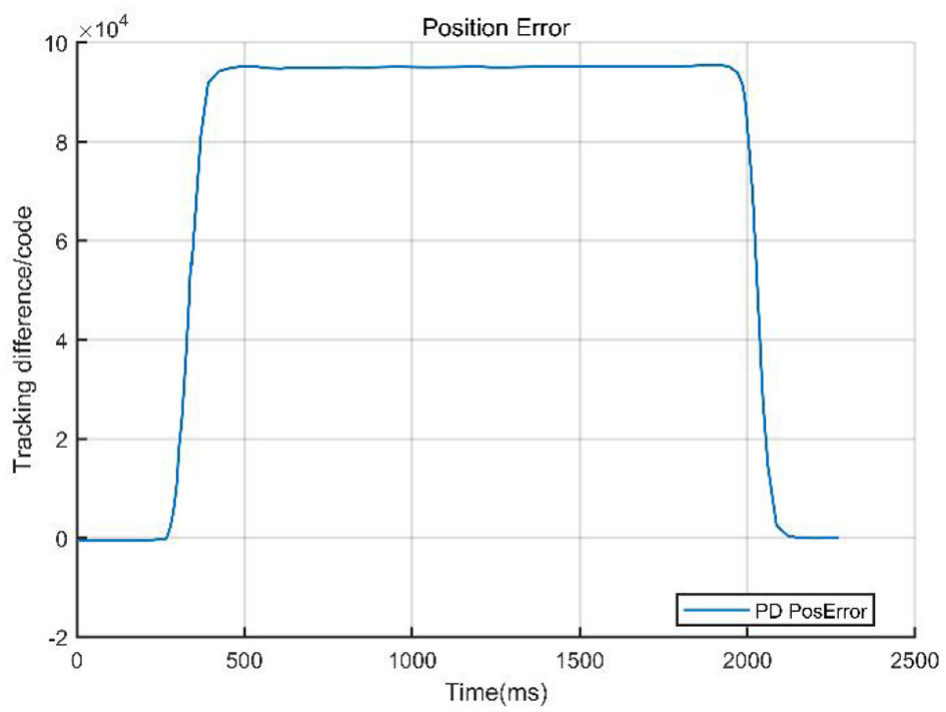
Tracking error of PD control.

**Figure 9:**
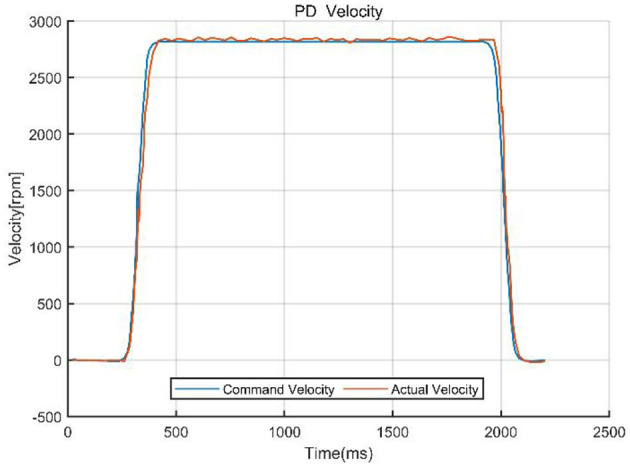
Command velocity vs. actual velocity with PD control.

To validate the effectiveness of the designed control method, we compared the PD control strategy with a PD-based composite control strategy that integrates singular perturbation theory. The core of the experiment involved quantitatively analyzing the system performance under both control architectures, focusing on key metrics such as system tracking error, velocity response curves, and velocity error. Figures 8, 9 visually demonstrate the system dynamic behavior using classical PD control, providing a benchmark for subsequent comparison with PD control combined with singular perturbation theory. In figures, the Tracking difference on the *Y*-axis represents the actual code drive value minus the planned code drive value, and its units can be expressed in PPR (Pulse Per Revolution).

**Figure 10:**
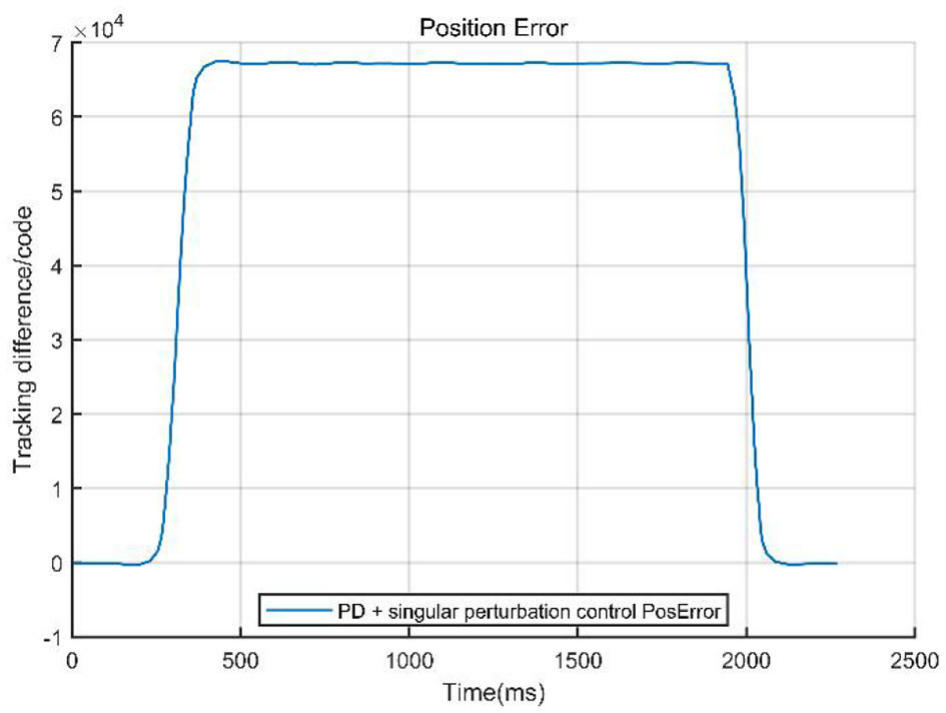
Tracking error of PD + singular perturbation control.

**Figure 11:**
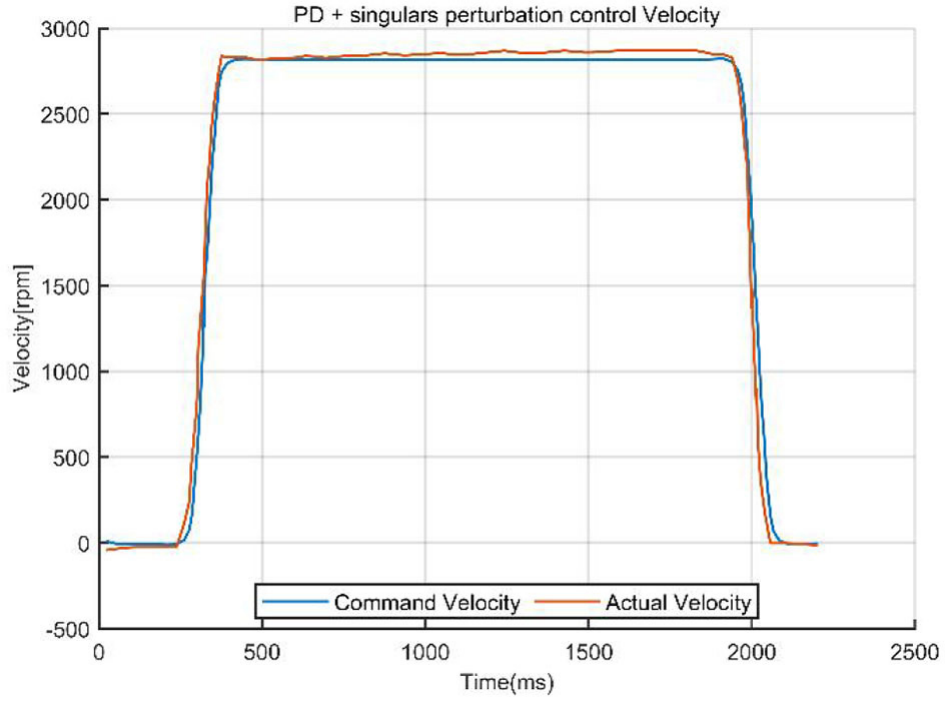
Command velocity vs. actual velocity with PD control + singular perturbation.

The system data curves for the PD combined with singular perturbation control are presented in Figures 10, 11.

**Figure 12:**
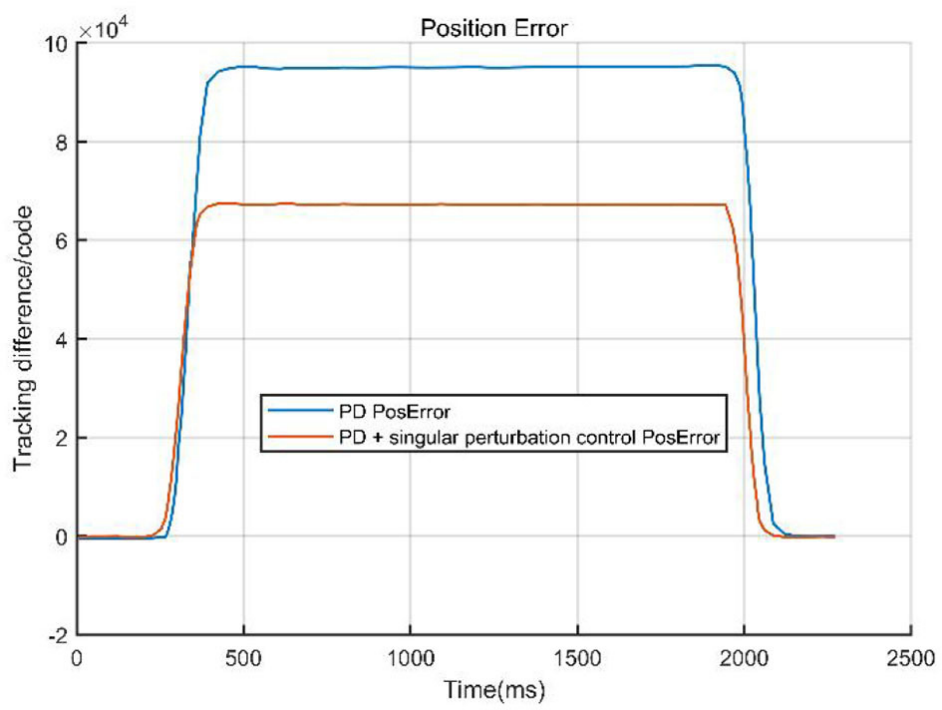
Compared curves of the two methods.

The comparison curves (Figure 12) indicate that a singular perturbation controller significantly enhances system tracking performance, reducing the tracking error by about one-third compared to using only a PD controller. As a critical step in evaluating the controller performance, experimental validation, through comparative data, demonstrates that the PD with singular perturbation control effectively reduces system tracking errors, ensuring more accurate trajectory tracking.

From Table 2, we can intuitively observe the advantages and disadvantages of the two control methods. The PD control excels in its simplicity and low computational cost, making it suitable for real-time applications in rigid systems. However, it struggles with multi-time-scale dynamics and external disturbances, leading to larger tracking errors and slower response times. In contrast, the PD + Singular Control (PD+SC) significantly improves response speed, tracking accuracy, and system robustness by effectively handling multi-time-scale dynamics. Nevertheless, it comes with increased complexity in controller design, higher computational demands, and potential errors due to model simplification. This comparison highlights the trade-offs between simplicity and performance in control system design.

**Table 2:** Compared methods.

**Method**	**Advantages**	**Limitations**
PD	1. Simple structure	1. Limited effectiveness in multi-time-scale systems
	2. Low computational cost	2. Difficulty in handling high-frequency dynamics
	3. Performs well in rigid systems	3. Larger tracking errors and slower response speed
PD+SC	1. Improves response	1. Complex controller design
	2. Effectively handles multi-time-scale dynamics	2. Higher computational cost
	3. Enhances system robustness	

The improvement in tracking error has significant practical implications for patient rehabilitation. By reducing tracking errors, the robot can more precisely follow the patient's movement intentions, minimizing unnecessary mechanical resistance or sudden adjustments. This enhanced precision makes the rehabilitation training feel more natural and comfortable for patients, reducing discomfort caused by inaccurate robot movements. Additionally, smaller tracking errors enable the robot to respond more accurately to the patient's actions during training, enhancing safety by preventing accidents due to control delays or errors. For example, if a patient suddenly stops or changes direction, the robot can adjust quickly and accurately, lowering the risk of injury. Furthermore, precise motion control allows patients to engage in more effective rehabilitation training. By providing consistent and accurate assistance, the robot helps patients better restore their motor functions, potentially accelerating the recovery process and shortening rehabilitation time. The experimental results allow for further adjustments and optimization of the control parameters to achieve the best control outcomes. This validates the theoretical design's correctness and provides valuable experience and data support for future engineering applications.

## 5 Conclusion

This paper focuses on developing a flexible lower limb rehabilitation robot, with an in-depth exploration of the construction process for motion control and singular perturbation algorithms. The classical DH method was employed to ensure the accuracy and practicality of the models. The study demonstrates a dynamic zero-load control algorithm to utilize a flexible lower-limb rehabilitation robotic. Take the friction term into the traditional dynamic equation and use the LuGre friction model for friction analysis to realize zero-force control. This approach enabled high-precision control of the end effector force in the robot, achieving effective force feedback management. In this paper, an optimal S-type planning with limited acceleration is proposed, and a complete algorithm for all possible trajectory shapes under various constraints is given. The trajectory planning algorithm proposed in this paper is more consistent with the actual motion performance of the robot. The limitations of traditional singular perturbation control methods in flexible joint robots were anal in control strategize. The rehabilitation robot employing the improved singular perturbation control strategy can rapidly respond to and adjust the applied external forces when the patient actively participates in training, significantly reducing the discomfort and stress risks perceived by the patient. This technology demonstrates significant control accuracy, system response speed, and operational safety advantages.

Looking ahead, we plan to expand further and optimize the findings of this research, including but not limited to improving the accuracy of the dynamic models, optimizing the efficiency of the control algorithms, and enhancing the robustness and adaptability of the system. Additionally, we will actively explore the application potential of this technology in other rehabilitation robot fields, such as upper limb and spinal rehabilitation, to benefit more patients in need of rehabilitation support.

## Data Availability

The raw data supporting the conclusions of this article will be made available by the authors, without undue reservation.
